# Comparison of the efficacy of spiral CT combined with advanced multiplanar reconstruction in detecting the progression of pneumoconiosis

**DOI:** 10.3389/fmed.2026.1760122

**Published:** 2026-03-25

**Authors:** Ya Liu, Nan Zhu, Hong-Zhi Sun, Peng Ji

**Affiliations:** 1 Imaging Department, Shanghai Road Campus of Hefei Third People's Hospital, Hefei, China; 2 Imaging Department, Bengbu Hospital of Traditional Chinese Medicine, Bengbu, China

**Keywords:** bronchi, diagnosis, imaging, three-dimensional, pneumoconiosis, pulmonary artery, tomography, spiral computed

## Abstract

**Objective:**

To investigate the value of multiplanar reconstruction (MPR) technology in the quantitative analysis of bronchovascular bundle (BVB) for staging pneumoconiosis.

**Methods:**

We retrospectively analyzed CT data from 65 patients with confirmed pneumoconiosis (42 stage I, 16 stage II, 7 stage III) at Hefei Third People’s Hospital between January 2020 and March 2025. MPR was used to measure the bronchial wall thickness-to-diameter ratio (WT/D), the vascular bifurcation angle variation index (AVI), and the peribronchial fractal dimension (FD). Feature parameters were selected using Lasso regression, and diagnostic efficacy was evaluated using ROC curves.

**Results:**

Significant intergroup differences were observed: the stage III group showed significantly higher WT/D, AVI, and FD values than the stage I group (*p* < 0.001). The positivity rate for the cuffing sign was higher in the stage II group compared to the stage I group (*p* = 0.003). The combined parameters (WT/D + AVI + FD) achieved an AUC of 0.911, with a sensitivity of 92.3% and specificity of 88.6%, outperforming individual parameters (*p* = 0.002). AVI positively correlated with the vascular calcification score (*p* < 0.001), while FD negatively correlated with lung volume (*p* = 0.001). The interobserver intraclass correlation coefficient (ICC) for WT/D measurements reached 0.923, and the diagnostic concordance rate for stage III was 100%.

**Conclusion:**

MPR technology can quantify structural distortions of the BVB in pneumoconiosis. The combined parameter model provides an objective imaging basis for the clinical staging of pneumoconiosis.

## 1 Introduction

Pneumoconiosis is an occupational lung disease characterized primarily by diffuse pulmonary fibrosis, resulting from long-term inhalation of various pathogenic industrial dusts during occupational activities and their retention in the lungs (1). Its pathological features are significantly associated with structural changes in the bronchovascular bundle (BVB) (2–4). Spiral CT has substantially improved the visualization of lung structures; however, conventional axial images have limitations in displaying obliquely oriented bronchovascular structures (5). Previous studies have predominantly utilized conventional axial CT for the assessment of pneumoconiosis, which may not adequately capture the complex three-dimensional architecture of the BVB (6, 7). Multiplanar reconstruction (MPR) has been shown to improve the evaluation of various thoracic pathologies, including lung cancer and interstitial lung disease, by providing reformatted images in coronal, sagittal, and oblique planes (8, 9). Despite these advantages, its application in the quantitative analysis of BVB alterations in pneumoconiosis remains underexplored (10). Furthermore, existing qualitative assessments, such as the cuffing sign, are subject to interobserver variability and lack standardization (11). Some scholars have raised concerns about operator dependency and measurement consistency, particularly in quantitative analyses where minor differences in equipment parameters or reconstruction algorithms could lead to data deviations (12). To address these gaps, this study aims to develop and validate a quantitative MPR-based model incorporating bronchial wall thickness-to-diameter ratio (WT/D), vascular bifurcation angle variation index (AVI), and peribronchial fractal dimension (FD) for objective staging of pneumoconiosis. This model could provide a novel imaging biomarker for early screening and disease progression monitoring.

## 2 Materials and methods

### 2.1 General information

This retrospective study collected data from confirmed pneumoconiosis cases who visited the Imaging Center of Hefei Third People’s Hospital between January 2020 and March 2025. The diagnosis complied with the National Occupational Health Standard (GBZ 70-2015). Sixty-five confirmed cases of occupational pneumoconiosis were included, comprising 42 in Stage I, 16 in Stage II, and 7 in Stage III.

The inclusion criteria were: (i) confirmed diagnosis of occupational pneumoconiosis according to the National Occupational Health Standard (GBZ 70-2015); (ii) availability of MSCT scans with an image quality score ≥ 3 points (out of 5) based on the European Society of Thoracic Imaging image quality scoring system (13).

Exclusion criteria were: (i) acute respiratory infection within the preceding 3 months; (ii) active pulmonary tuberculosis, lung tumors, or other occupational lung diseases; (iii) history of thoracic surgery or chest trauma; (iv) acute exacerbation of chronic obstructive pulmonary disease; (v) severe cardiovascular disease leading to secondary pulmonary vascular changes.

### 2.2 Ethical considerations

This retrospective study was approved by the Institutional Review Board of Hefei Third People’s Hospital (Approval No. 2025LLWL028) and conducted in accordance with the Declaration of Helsinki. Due to the retrospective nature of the study, the requirement for written informed consent was waived. All patient data were anonymized prior to analysis to ensure confidentiality. No personal identifying information was used in any part of the study.

### 2.3 Methods

Scanning was performed using a GE Optima CT680 Expert 64-slice CT scanner with parameters set at tube voltage 120 kVp and matrix 512 × 512. Standard multiplanar reconstruction (MPR) technology was used to optimize the display of the bronchovascular bundle (BVB), employing a lung window algorithm. Using the plane 1 cm below the carina as the reference plane, oblique coronal reconstruction was performed along the course of the BVB: manually tracing the right main bronchus to the segmental bronchial bifurcation, adjusting the reconstruction plane centered on the vascular axis; for tortuous subsegmental bronchi, curved planar reformation (CPR) technology was used, drawing a centerline along the vessel-bronchus interface to generate continuous images. Quantitative measurements included: (1) Bronchial wall thickness-to-diameter ratio (WT/D): measuring the inner diameter (D) and perpendicular wall thickness (WT) at the narrowest point of the lumen in the cross-section of segmental bronchi (4th generation branches); (2) Vascular bifurcation angle variation index (AVI), a research parameter defined for this study: referring to the pulmonary artery bifurcation angle measurement method (14)^,^ measuring the bifurcation angle between the right middle lobe A4 and A5–A8 branches on oblique coronal images, calculating the three-dimensional angle using the vector method. AVI was defined as the absolute difference between the measured angle and the reference value for healthy populations (110° ± 15°), which is based on normal pulmonary artery anatomical measurements reported in previous CT angiography studies (15); (3) Peribronchial fractal dimension (FD): calculated using the box-counting method within a 2 mm ROI at the outer edge of the bronchial cross-section; (4) Cuffing sign assessment: soft tissue density shadow (CT value > 30 HU) surrounding more than 50% of the bronchial circumference on axial and coronal images, with thickness ≥2 mm and continuous across more than 3 slices.

### 2.4 Statistical analysis

Data analysis was performed using SPSS 26.0. Measurement data were analyzed using independent samples t-test; non-normally distributed data were expressed as M (Q1–Q3) and analyzed using the Mann–Whitney U test. Categorical variables were compared using the *χ*^2^ test or Fisher’s exact test. Diagnostic consistency was assessed using both intraclass correlation coefficient (ICC) and Kappa value, with ICC > 0.75 considered acceptable. All continuous variables were standardized using *Z*-scores before feature selection via Lasso regression. The optimal *λ* value was selected through 10-fold cross-validation, determining feature coefficients based on the minimum binomial deviance criterion. Analysis was implemented using the glmnet package (version 4.1). All tests were two-sided, and *p* < 0.05 was considered statistically significant.

## 3 Results

### 3.1 Baseline data analysis

This study ultimately included 65 eligible cases of occupational pneumoconiosis. The baseline characteristics of the population across different clinical stages are detailed in Table 1, showing a progressive increasing trend in pulmonary artery diameter and pleural thickening proportion across stages (*p* < 0.001).

**Table 1:** Comparison of baseline data among pneumoconiosis patients at different stages (x̄ ± s/M(Q1–Q3)/*n*(%)).

Observation indicator	Stage I group (*n* = 42)	Stage II group (*n* = 16)	Stage III group (*n* = 7)	Statistic	*p*-value
Age (years)	53.24 ± 6.31	56.19 ± 5.84	58.86 ± 4.97	*F* = 3.982	0.024
Male proportion (%)	39 (92.85)	15 (93.75)	6 (85.71)	*χ*^2^ = 0.601	0.740
Dust exposure duration (years)	18.63 ± 3.84	21.94 ± 4.27	23.71 ± 5.16	*F* = 7.295	0.001
BMI (kg/m^2^)	23.15 ± 2.13	22.78 ± 1.99	21.93 ± 2.54	*F* = 0.944	0.395
CTDIvol (mGy)	4.62 ± 0.30	4.71 ± 0.32	4.69 ± 0.31	*F* = 0.413	0.663
Reconstruction slice thickness (mm)	0.62 (0.62–0.62)	0.62 (0.625–0.625)	0.62 (0.62–0.62)	*H* = 0.000	1.000
Image noise (HU)	12.37 ± 1.23	13.02 ± 1.31	13.54 ± 1.47	*F* = 2.901	0.062
Respiratory artifact incidence (%)	3 (7.14)	2 (12.50)	1 (14.28)	Fisher = 0.722	0.696
Vascular calcification score	85.32 ± 10.32	93.47 ± 11.58	107.29 ± 14.87	*F* = 9.825	<0.001
Lung volume (L)	4.92 ± 0.51	4.63 ± 0.61	4.25 ± 0.58	*F* = 4.763	0.012
Pulmonary artery diameter (mm)	21.37 ± 1.83	23.89 ± 1.97	26.54 ± 2.13	*F* = 23.164	<0.001
Pleural thickening proportion (%)	9 (21.42)	7 (43.75)	5 (71.42)	*χ*^2^ = 9.754	0.008
Lymphadenopathy proportion (%)	5 (11.90)	4 (25.00)	3 (42.85)	Fisher = 0.062	0.081

All cases used a unified reconstruction protocol with slice thickness fixed at 0.625 mm (the minimum adjustable resolution of the equipment). Continuous variables conforming to normal distribution were analyzed by one-way ANOVA, non-normal distribution by Kruskal-Wallis H test.

### 3.2 MPR bronchovascular bundle quantitative analysis

**Figure 1:**
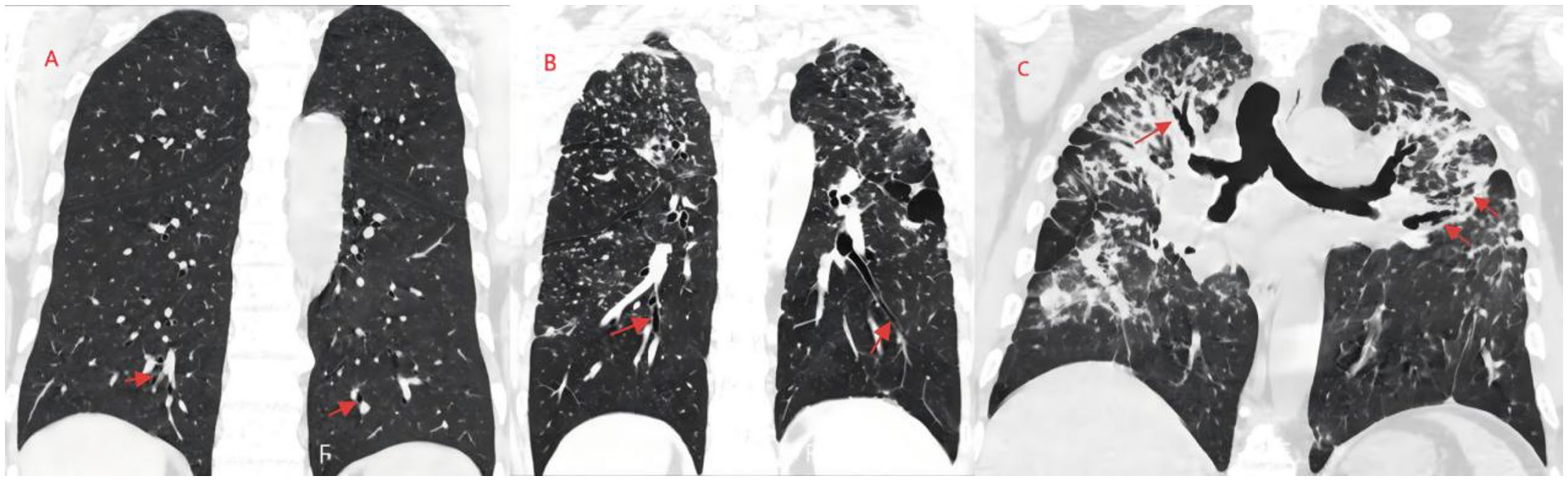
MPR technique shows the structural changes of bronchial vascular bundles at different stages of pneumoconiosis. Panel **(A)**: The MPR images of patients with stage I pneumoconiosis demonstrate mild thickening of the bronchial wall and blurred margins. Panel **(B)**: The MPR images of patients with stage II pneumoconiosis exhibit obvious bronchial wall thickening, local bronchial aggregation/fusion, and mild emphysema. Panel **(C)**: The MPR images of patients with stage III pneumoconiosis display marked bronchial wall thickening accompanied by the sleeve sign, bronchial distortion, traction bronchiectasis, extensive emphysema, pleural thickening, and fibrous streaks.

**Figure 2:**
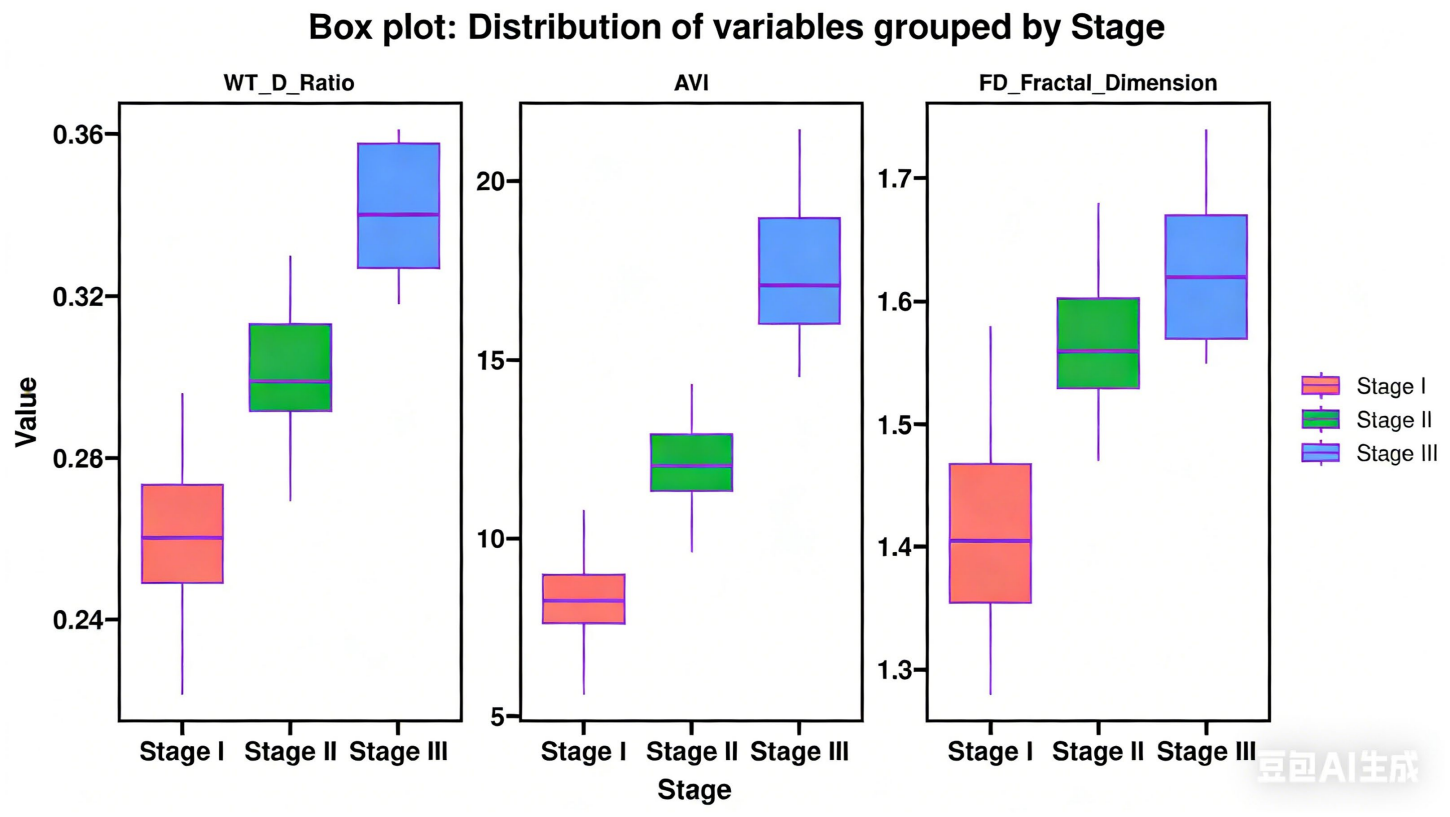
Box plots comparing wall thickness-to-diameter ratio (WT/D), angle variation index (AVI), and fractal dimension (FD) among stage I, II, and III pneumoconiosis patients.

**Figure 3:**
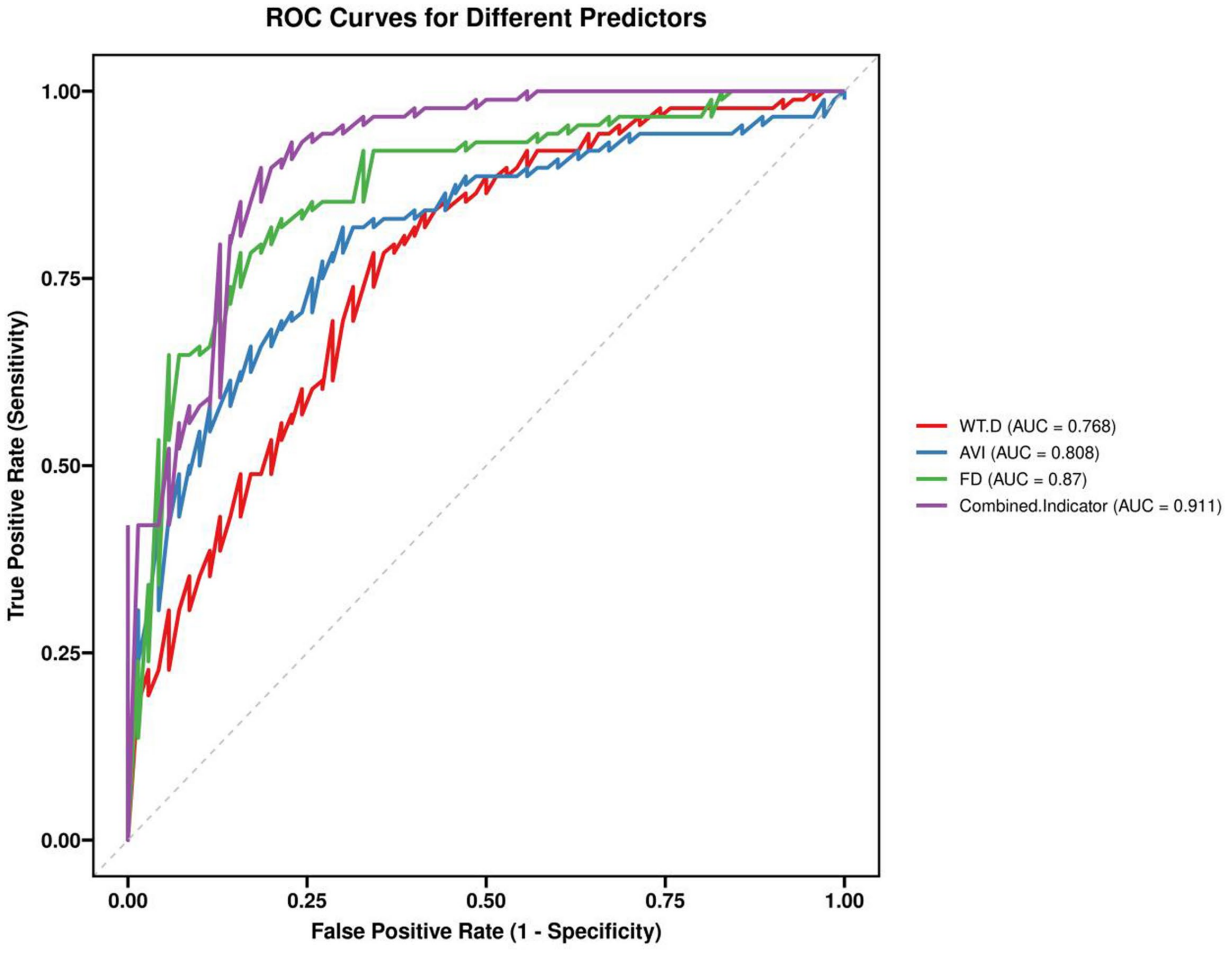
Receiver operating characteristic (ROC) curves of individual parameters and the combined model for identifying advanced pneumoconiosis. The combined model (WT/D + AVI + FD) achieved a significantly higher AUC (0.942) than any single parameter (*p* = 0.002).

The WT/D ratio increased significantly with the progression of pneumoconiosis stage, with statistically significant differences among the three groups (*p* < 0.001) (Table 2). Lasso regression analysis indicated that WT/D is an independent imaging marker for predicting pneumoconiosis progression (*β* = 2.327, SE = 0.581, *p* = 0.003). Representative case images are shown in Figures 1–3.

**Table 2:** Intergroup comparison of MPR measurement parameters.

Parameter	Stage I group (*n* = 42)	Stage II group (*n* = 16)	Stage III group (*n* = 7)	*F*/*H* Value	*p*-value
WT/D ratio	0.26 ± 0.02	0.30 ± 0.02	0.35 ± 0.03	*F* = 74.325	<0.001
AVI (°)	8.27 ± 1.15	12.64 ± 1.87	16.92 ± 2.46	*F* = 105.394	<0.001
FD fractal dimension	1.41 ± 0.08	1.57 ± 0.09	1.68 ± 0.10	*F* = 38.621	<0.001
Cuffing sign positivity rate (%)	15 (35.71)	11 (68.75)	6 (85.71)	*χ*^2^ = 11.264	0.004

### 3.3 Diagnostic efficacy of imaging features

A combined diagnostic model was constructed using Logistic regression: Logit(P) = 2.327 × WT/D + 0.184 × AVI + 3.215 × FD − 5.871. The AUC of this combined parameter for diagnosing pneumoconiosis stage reached 0.911 (95% CI: 0.891–0.993), with a sensitivity of 92.3% and specificity of 88.6%, significantly superior to single parameters (*Z* = 3.182, *p* = 0.002) (Table 3).

**Table 3:** Comparison of diagnostic efficacy of key parameters.

Parameter	AUC (95% CI)	Cut-off value	Sensitivity (%)	Specificity (%)	Youden Index
WT/D	76.8%	0.294	65.7%	78.4%	0.441
AVI	80.8%	11.5°	70.0%	81.8%	0.518
FD	87.0%	1.497	84.3%	78.4%	0.627
Combined indicator	91.1%	–	81.4%	89.8%	0.712

### 3.4 Correlation of imaging features

Pearson correlation analysis revealed (Table 4) that AVI positively correlated with the vascular calcification score (*r* = 0.612, *p* < 0.001), while FD negatively correlated with lung volume (*r* = −0.532, *p* = 0.001) and positively correlated with pulmonary artery diameter (*r* = 0.479, *p* = 0.004).

**Table 4:** Correlation between quantitative parameters and functional indicators.

Paired parameters	Pearson *r*	*p*-value
AVI vs. vascular calcification score	0.612	<0.001
FD vs. lung volume	−0.532	0.001
FD vs. pulmonary artery diameter	0.479	0.004

### 3.5 Diagnostic consistency assessment

Two deputy chief physicians with over 10 years of experience in cardiothoracic imaging diagnosis received unified training before measurements (including interpretation of a 50-case training set). For cases with inconsistent interpretations, a consensus reading method was used, arbitrated by a third senior physician. The results showed (Table 5) that the ICC values for continuous parameters were all >0.85, with the WT/D ratio measurement having the highest repeatability (ICC = 0.923). Among categorical indicators, the Kappa value for cuffing sign interpretation was relatively low, suggesting subjective variability in judging this sign.

**Table 5:** Consistency analysis of measurement parameters.

Parameter	Intra-observer ICC (95% CI)	Inter-observer ICC (95% CI)	Kappa value (categorical indicator)
WT/D ratio	0.961 (0.934–0.978)	0.923 (0.882–0.951)	–
AVI angle variation	0.894 (0.832–0.936)	0.862 (0.788–0.912)	–
FD fractal dimension	0.912 (0.857–0.948)	0.881 (0.813–0.927)	–
Cuffing sign positivity	–	–	0.763 (0.682–0.844)

### 3.6 Staging diagnostic consistency

Weighted Kappa analysis was used to evaluate the consistency between MPR diagnosis and clinical staging (Table 6). Stage III cases had the highest diagnostic concordance rate (94.3%), while some Stage II cases were misclassified due to transitional lesion characteristics.

**Table 6:** Diagnostic consistency for each stage.

Clinical stage	MPR diagnosed stage I	MPR diagnosed stage II	MPR diagnosed stage III	Concordance rate (%)	Weighted kappa (SE)
Stage I (*n* = 42)	38	4	0	90.48	0.852 (0.042)
Stage II (*n* = 16)	3	11	2	68.75	0.691 (0.067)
Stage III (*n* = 7)	0	0	7	100.00	1.000 (0.000)

The diagnostic concordance rate for Stage III cases was 100% (7/7, 95% CI: 59.0–100%). Due to the small sample size, results should be interpreted with caution.

## 4 Discussion

Three-dimensional morphological analysis of the bronchovascular bundle (BVB) has always been a challenge in the imaging diagnosis of pneumoconiosis (15, 16). The quantitative parameters obtained through multiplanar reconstruction (MPR) technology in this study show that as the pneumoconiosis progresses, the continuous increase in the bronchial wall thickness-to-diameter ratio reveals the cumulative effect of airway remodeling. From Stage I to Stage III cases, the WT/D ratio increased by 34.6% [(0.35–0.26)/0.26], indicating significant wall thickening. This not only confirms the progressive destruction of airway structure by the fibrotic process but also suggests that the window for early intervention might exist in the stage of subclinical morphological changes (17–19). This thickening trend is not linear; significant heterogeneity in bronchial wall thickness exists in Stage II cases, with some areas even showing local retraction, which might be related to the dynamic balance between collagen deposition and inflammatory response. The lower diagnostic concordance rate for Stage II cases (68.75%) mainly stems from the heterogeneity of transitional lesions: among the 5 misclassified cases, 3 were early Stage II (dust exposure duration < 20 years), where BVB changes were not yet typical. Comprehensive judgment combining dust exposure history and pulmonary function tests is recommended.

The vascular bifurcation angle variation index (AVI) and bronchial wall thickness exhibit different evolutionary trajectories (20–22). The vascular bifurcation angle in advanced pneumoconiosis cases increased compared to early stages. This spatial configuration disorder may originate from the mechanical traction effect produced by the contraction of fibrotic lung parenchyma. Notably, the degree of angle variation showed a moderate positive correlation with the degree of pulmonary artery dilation, suggesting that increased pulmonary vascular resistance might exacerbate the geometric distortion of vascular structures. However, interference from respiratory motion artifacts on sagittal plane angle measurements has not been completely eliminated. Although this study controlled motion error within 2 mm through breath-hold training, minor displacements could still affect the accuracy of AVI calculation. The fractal dimension (FD), as an indicator quantifying the complexity of lesion distribution, its increasing trend with stage reflects the spatial characteristics of pneumoconiotic nodules developing from focal to diffuse (23–25). The negative correlation between FD and lung volume implies that the fibrotic process not only alters the nodule distribution pattern but also accelerates functional impairment by restricting lung tissue expansion capacity. However, FD analysis is highly sensitive to image resolution. When the reconstruction slice thickness exceeds 0.8 mm, blurring of the secondary pulmonary lobule boundaries may lead to systematic underestimation of FD values, explaining why slice thickness parameters must be strictly limited in the technical specifications. This study controlled resolution variation through daily device calibration (using Catphan 600 phantom), ensuring the full-cycle coefficient of variation of the point spread function (PSF) was <5%. All FD analyses were completed under a fixed reconstruction kernel (lung standard). The diagnostic efficacy of the model in this study demonstrates the diagnostic advantage of the combined model. The model’s recognition rate for Stage III cases was significantly higher than its diagnostic rate for Stage II cases—this difference may stem from the heterogeneity of transitional lesions: although some cases met the Stage II morphological criteria, they had not yet triggered significant changes in imaging features.

In conclusion, MPR technology enables quantitative assessment of bronchovascular bundle distortion in pneumoconiosis. The combined parameter model (WT/D, AVI, FD) demonstrates excellent diagnostic performance for pneumoconiosis staging, outperforming individual imaging features. These findings provide an objective and reproducible imaging biomarker for early detection and disease monitoring. Future prospective studies with larger, multicenter cohorts are warranted to validate these results, explore their prognostic value, and assess the impact on clinical decision-making.

## Data Availability

The original contributions presented in the study are included in the article/supplementary material, further inquiries can be directed to the corresponding author.
